# Supplementary material for: The interactome of intact mitochondria by cross-linking mass spectrometry provides evidence for coexisting respiratory supercomplexes
Source: Mol Cell Proteomics. 2017 Dec 8;17(2):216–32. doi: 10.1074/mcp.RA117.000470 (PMC5795388; doi:10.1074/mcp.RA117.000470)
Supplement: Supplemental Data [file supp_RA117.000470_133922_0_supp_23978_fzffwf.zip › spectra_annotation/mito_spectra_annotation/283-1-12-1-62-1.pdf]

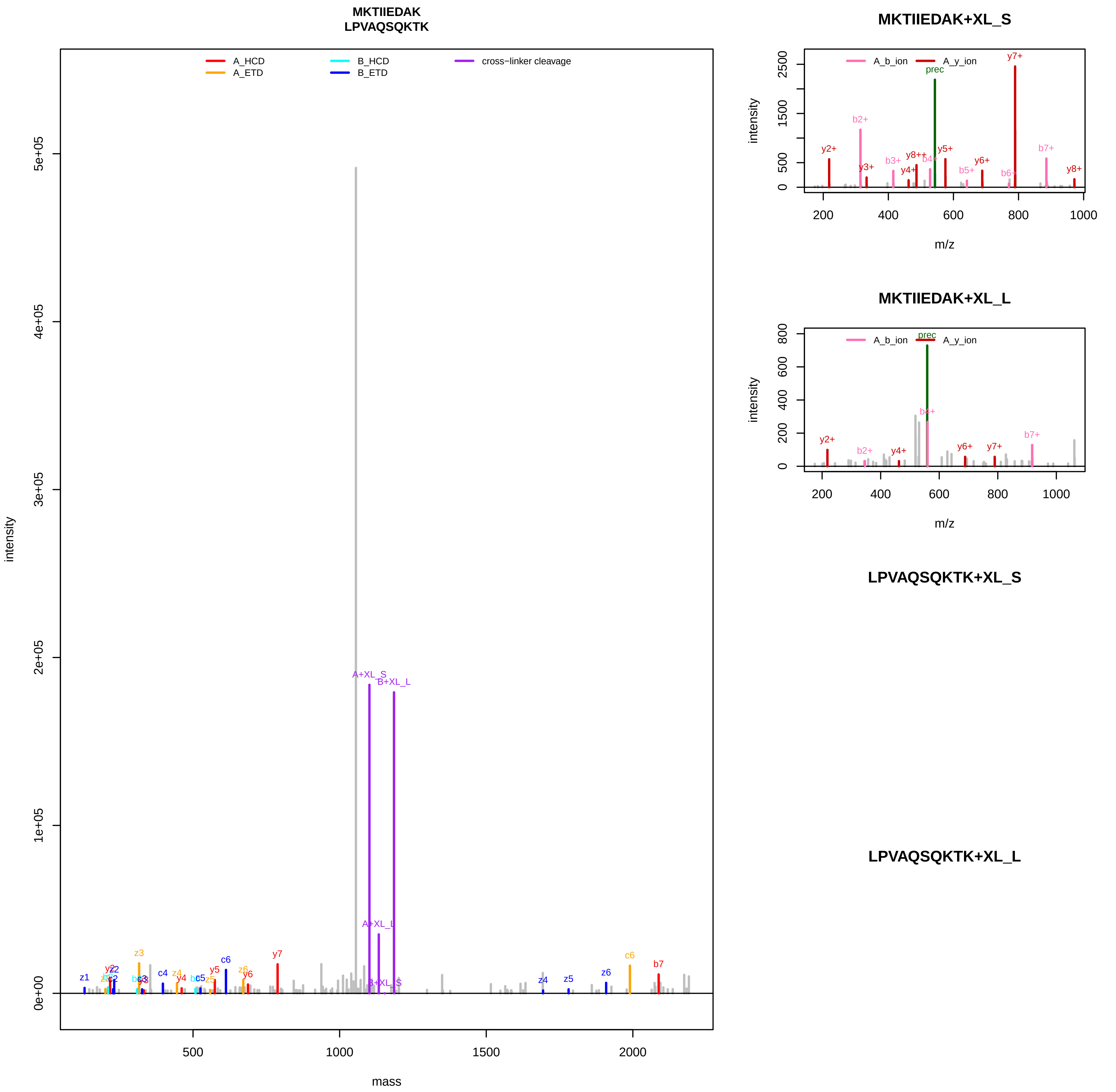

MKTIIEDAK
LPVAQSQKTK
A_HCD
A_ETD
B_HCD
B_ETD
cross−linker cleavage
intensity
mass
A+XL_S
B+XL_L
A+XL_L
B+XL_S
z1
y2
z2
c2
z3
c3
y3
c4
z4
y4
c5
z5
y5
c6
z6
y6
y7
z4
z5
z6
c6
b7
MKTIIEDAK+XL_S
A_b_ion
A_y_ion
prec
y7+
b2+
y2+
y3+
b3+
y8++
y4+
b4+
y5+
b5+
y6+
b6+
b7+
y8+
m/z
MKTIIEDAK+XL_L
LPVAQSQKTK+XL_S
LPVAQSQKTK+XL_L
